# Loss of PHF6 leads to aberrant development of human neuron-like cells

**DOI:** 10.1038/s41598-020-75999-2

**Published:** 2020-11-04

**Authors:** Anna Fliedner, Anne Gregor, Fulvia Ferrazzi, Arif B. Ekici, Heinrich Sticht, Christiane Zweier

**Affiliations:** 1 Institute of Human Genetics, Friedrich-Alexander-University Erlangen-Nürnberg, 91054 Erlangen, Germany; 2 Institute of Biochemistry, Friedrich-Alexander-University Erlangen-Nürnberg, 91054 Erlangen, Germany; 3 Department of Human Genetics, Inselspital, Bern University Hospital, University of Bern, Freiburgstrasse 15, 3010 Bern, Switzerland

**Keywords:** Genetics research, Developmental disorders

## Abstract

Pathogenic variants in PHD finger protein 6 (PHF6) cause Borjeson–Forssman–Lehmann syndrome (BFLS), a rare X-linked neurodevelopmental disorder, which manifests variably in both males and females. To investigate the mechanisms behind overlapping but distinct clinical aspects between genders, we assessed the consequences of individual variants with structural modelling and molecular techniques. We found evidence that de novo variants occurring in females are more severe and result in loss of *PHF6*, while inherited variants identified in males might be hypomorph or have weaker effects on protein stability. This might contribute to the different phenotypes in male versus female individuals with BFLS. Furthermore, we used CRISPR/Cas9 to induce knockout of *PHF6* in SK-N-BE (2) cells which were then differentiated to neuron-like cells in order to model nervous system related consequences of *PHF6* loss. Transcriptome analysis revealed a broad deregulation of genes involved in chromatin and transcriptional regulation as well as in axon and neuron development. Subsequently, we could demonstrate that PHF6 is indeed required for proper neuron proliferation, neurite outgrowth and migration. Impairment of these processes might therefore contribute to the neurodevelopmental and cognitive dysfunction in BFLS.

## Introduction

Variants in the gene encoding PHD finger protein 6 (*PHF6* [MIM: 300414]) have been identified to cause Borjeson–Forssman–Lehmann syndrome (BFLS [MIM: #301900]), an X-linked syndromic neurodevelopmental disorder (NDD)^1,2^, affecting both male and female individuals. In males, it is characterized by mild to severe intellectual disability (ID), epilepsy, a distinct facial gestalt, obesity and hypogonadism^1,3,4^. In these families, female carriers are usually unaffected or present with mild symptoms, only^2,3^. Recently, de novo variants in *PHF6* were identified in affected females with NDDs, partly overlapping with BFLS in males but additionally with very distinct phenotypic aspects^4–7^. They presented with mild to severe ID, a characteristic facial gestalt, finger and toe anomalies, irregularly shaped teeth and oligodontia^4,5^.

The mutational spectrum encompasses truncating and missense variants in *PHF6* in both genders, thus not allowing obvious delineations of a genotype–phenotype correlation. Linear skin pigmentation in many of the affected females in combination with skewed X-inactivation in blood versus random X-inactivation in fibroblasts pointed to a functional mosaicism in these individuals^4^. This might contribute to the manifestation of a relatively severe, distinct NDD in females.

*Phf6* expression levels are particularly high in the developing mouse brain, and it is also highly expressed in developing facial structures (pharyngeal arches and the nasal process) and in limb buds^8^. The expression pattern is therefore in line with the neurodevelopmental phenotype, facial dysmorphism and finger/toe anomalies in affected human individuals^8^.

*PHF6* encodes a protein with two extended atypical PHD-like zinc finger domains (ePHD), two nuclear and one nucleolar localization sequences^2^. Due to the DNA-binding domains^9^ and its co-localization with transcriptionally active euchromatin in the nucleus^8^, a role as transcriptional regulator and/or chromatin remodeler has been suggested (reviewed in^10^). Furthermore, PHF6 functions as a histone reader recognizing H2BK12 acetylation via the extended part of its C-terminal PHD, and additionally as a histone writer/E3 ubiquitin ligase by triggering H2BK120 ubiquitination through the extended part of N-terminal ePHD during trophectoderm development^11^.Several protein-interaction partners of PHF6 have been identified, most of them indeed involved in transcriptional and chromatin regulation. Interaction of PHF6 with the PAF1 transcription elongation complex^12,13^ might play a role in driving neuronal migration^12^, and by interaction with upstream binding factor UBTF^12,14^ (MIM: 600673), it might be involved in ribosomal DNA transcription^14,15^. In addition, PHF6 was demonstrated to interact with histone deacetylase 1 (HDAC1 [MIM: 601241])^16^ and the ATP-dependent chromatin remodelling NuRD complex^16^. More recently it was shown that PHF6 is required to maintain a precise chromatin landscape in leukemia cells^17^. It is also regulating transcriptional networks by its chromatin-binding capacities, dependent on the nutritional state^18^.

Furthermore, a role of PHF6 deficiency in impaired cell proliferation, cell cycle arrest and increased DNA damage at the rDNA locus has been controversially discussed^14,15,17,19^.

We could now show that both truncating and missense variants in females result in loss of PHF6 while structural modelling indicated that variants identified in males might be less severe. Furthermore, RNA sequencing on human neuron-like cells upon CRISPR/Cas9 mediated knockout of *PHF6* revealed a broad deregulation of genes enriched for chromatin organization, transcription, neuron generation and nervous system development. Analysis of these *PHF6* knockout cells showed altered neurite outgrowth, proliferation and migration. Our observations therefore implicate these processes in the pathomechanism of BFLS.

## Results

### Predicted destabilizing effects of missense variants in *PHF6*

In male and female individuals with BFLS both truncating and missense variants in *PHF6* have been reported as pathogenic^1,4,6,7,20–29^, though missense variants seem to be significantly more frequent in males (13 independent families versus 1 individual) and truncating variants (7 independent families versus 11 cases/families) more frequent in females (p < 0.005, Fisher’s exact test).

**Figure 1:**
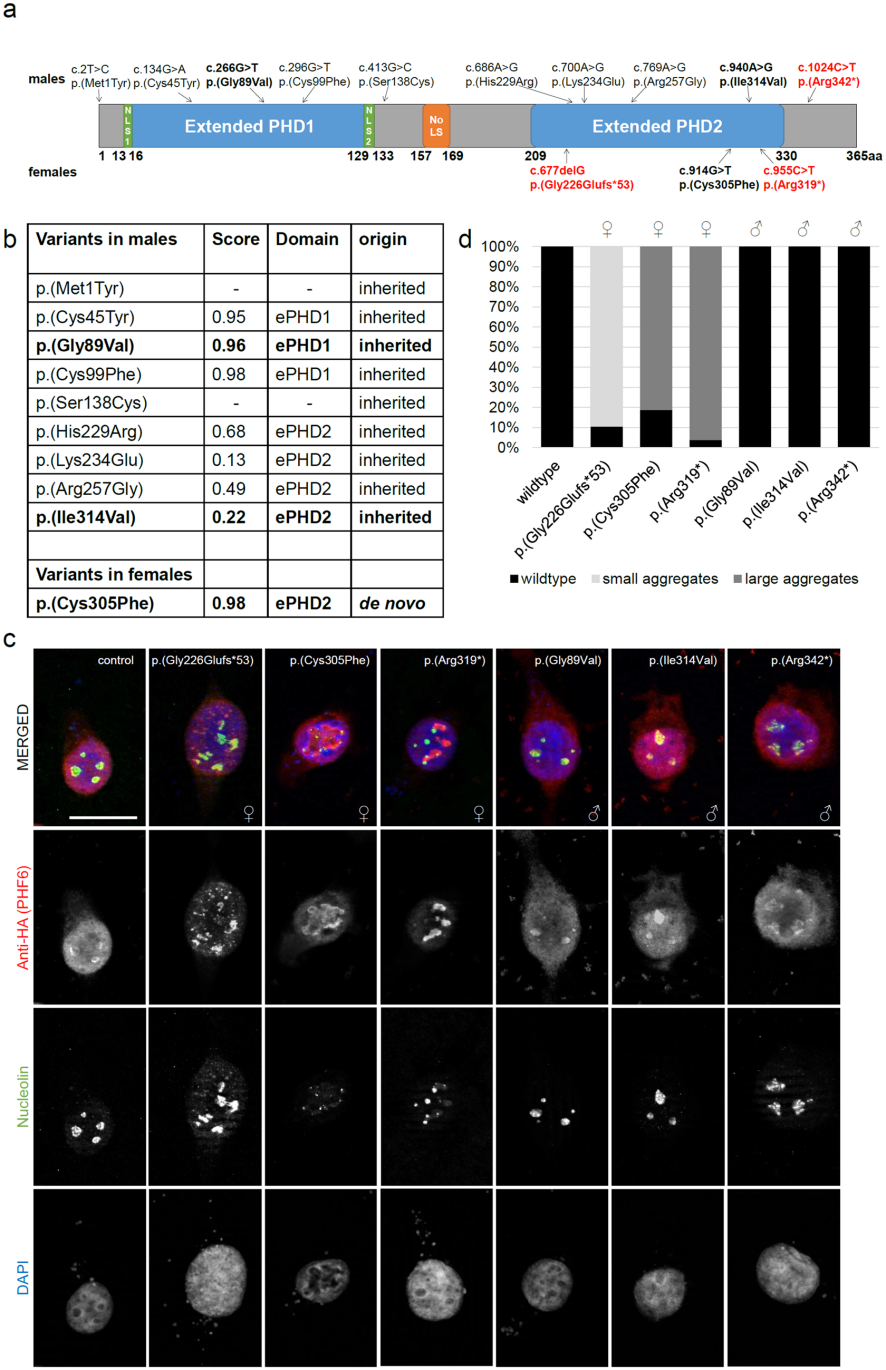
Overexpression analysis of variants in *PHF6* from females vs males. (**a**) Protein scheme of PHF6 (Q8IWS0) with all missense variants previously published in male and female individuals^2,4,6,21,23,24^ as well as three selected truncating variants^2,4^ analysed via immunofluorescence coloured in red. Analysed variants (immunofluorescence) are printed in bold. Coding domains (according to uniprot^91^) are displayed in colour. *NLS* nuclear localization domain, *NoLS* nucleolar localization domain. (**b**) Table including all published missense variants occurring in male and female individuals, their VIPUR score, their localization in regard to ePHD and their origin (inherited/de novo). Variants p.(Met1Tyr) and p.(Ser138Cys) do not reside in a domain and cannot be modelled. Analysed variants (immunofluorescence) are printed in bold. *ePHD* extended atypical PHD-like zinc finger domain, 1 = N-terminal, 2 = C-terminal. (**c**) Immunofluorescence of HEK-293 cells transiently transfected with HA-tagged wildtype or mutant *PHF6* co-stained with nucleolin (NCL). While wildtype PHF6 was expressed in the nucleoplasm as well as the nucleolus co-localizing with NCL, all three variants occurring in females led to an accumulation of PHF6 into aggregates, partly co-localizing with NCL. The truncating variant c.677delG (p.(Gly226Glufs*53)) resulted in small and more aggregates in the nucleus, and the two variants in the C-terminal ePHD resulted in large PHF6 aggregates while the expression of aberrant PHF6 in the nucleoplasm is reduced. In contrast, the three variants occurring in males resembled the wildtype with PHF6 expression in both the nucleoplasm and the nucleolus, co-localizing with NCL. Per variant, at least 40 cells were analyzed. Scale bar depicts 20 µm. Cells were stained with rabbit anti-HA (H6908, Sigma-Aldrich, 1:75) and mouse anti-nucleolin (39–6400, Thermo Fisher Scientific, 1:200). (**d**) Quantification of nuclear aggregates. The number of cells with or without aberrant large or small PHF6 aggregates in the nucleus was quantified for each variant and the wildtype. At least 40 cells were evaluated for each variant. ♀, variant identified in a female individual; ♂, variant identified in a male individual.

From literature we retrieved nine missense variants in *PHF6* identified in males^2,6,21,23,24^ and one missense variant identified in a female with BFLS^4^. Apart from two variants located close to the N-terminal ePHD, all missense variants were residing in either the N-terminal or the C-terminal ePHD (Fig. 1a). Variants within domains were assessed regarding their consequences on PHF6 protein structure using the program VIPUR^30^. Interestingly, missense variants from males that where located in the N-terminal ePHD had very high scores above 0.9, indicating strong destabilizing effects on the protein structure, while missense variants from males located in the C-terminal ePHD had significantly lower scores, indicating less severe effects on protein structure (Fig. 1a,b). In contrast, the only missense variant reported in females, so far, is located in the C-terminal ePHD and had a very high score of 0.98, indicating a stronger destabilizing effect on the protein structure than the variants from males residing in the same ePHD (Fig. 1a,b).

In silico analyses therefore predict variable destabilizing effects of missense variants dependent on their location.

### Localization differences of mutant PHF6

To investigate if *PHF6* variants might result in altered subcellular localization in vitro, we investigated the consequences of two missense variants identified in males [one from each ePHD, respectively (c.266G>T, p.(Gly89Val); c.940A>G, p.(Ile314Val))^6,23^] and the only missense variant reported in females (c.914G>T, p(Cys305Phe))^4^ in the C-terminal ePHD. Additionally, we analyzed one C-terminal truncating variant reported in males (c.1024C>T, p.(Arg342*))^2^ and two truncating variants reported in females in the C-terminal ePHD (c.677delG, p.(Gly226Glufs*53); c.955C>T, p.(Arg319*))^4^ (Fig. 1a). We overexpressed HA-tagged wildtype or mutant *PHF6* in HEK-293 cells. As reported before^2^, wildtype PHF6 showed an even distribution in nucleoplasm and nucleolus, as demonstrated by co-staining with nucleolin (NCL) (Fig. 1c). The two missense and one truncating variants identified in males behaved similar to the wildtype, with PHF6 and NCL co-localizing in the nucleolus (Fig. 1c). Additionally, protein expression levels were comparable to wildtype expression for the missense variant in the C-terminal ePHD and slightly reduced for the missense variant in the N-terminal ePHD (46% residual protein) or the truncating variant (88% residual protein) (Supplementary Fig. S1). In contrast, one missense and two truncating variants identified in females were still expressed in the nucleus, but accumulated in aggregates (Fig. 1c). Aggregates resulting from the early truncating p.(Gyl226Glyfs*53) variant appeared rather small. In cells overexpressing the more C-terminal truncating and the missense variant, larger aggregates were observed, while expression of mutant PHF6 in the nucleoplasm was reduced. Also co-localization with NCL was more variable, with the early truncating variant partly co-localizing with NCL in the nucleolus, and the more C-terminal variant barely or not co-localizing with NCL (Fig. 1c). More than 80% of analyzed cells (at least 40 cells/variant) presented with the same aggregate expression pattern. Quantification is provided in Fig. 1d. Furthermore, mutant PHF6 protein levels were markedly reduced in comparison to wildtype (3–7% residual protein), suggesting impairment of either mRNA stability, transcription, translation or protein stability (Supplementary Fig. S1).

Taken together, overexpression of *PHF6* carrying variants identified in females led to altered subcellular localization of PHF6 with formation of aggregates and markedly decreased protein levels, while PHF6 containing variants identified in males behaved more similar to wildtype PHF6. Though tested in a rather “artificial” situation by overexpression, variants identified in females or males behaved differently with more severe consequences for “female” variants.

### Pathogenic variants in females result in loss of PHF6

**Figure 2:**
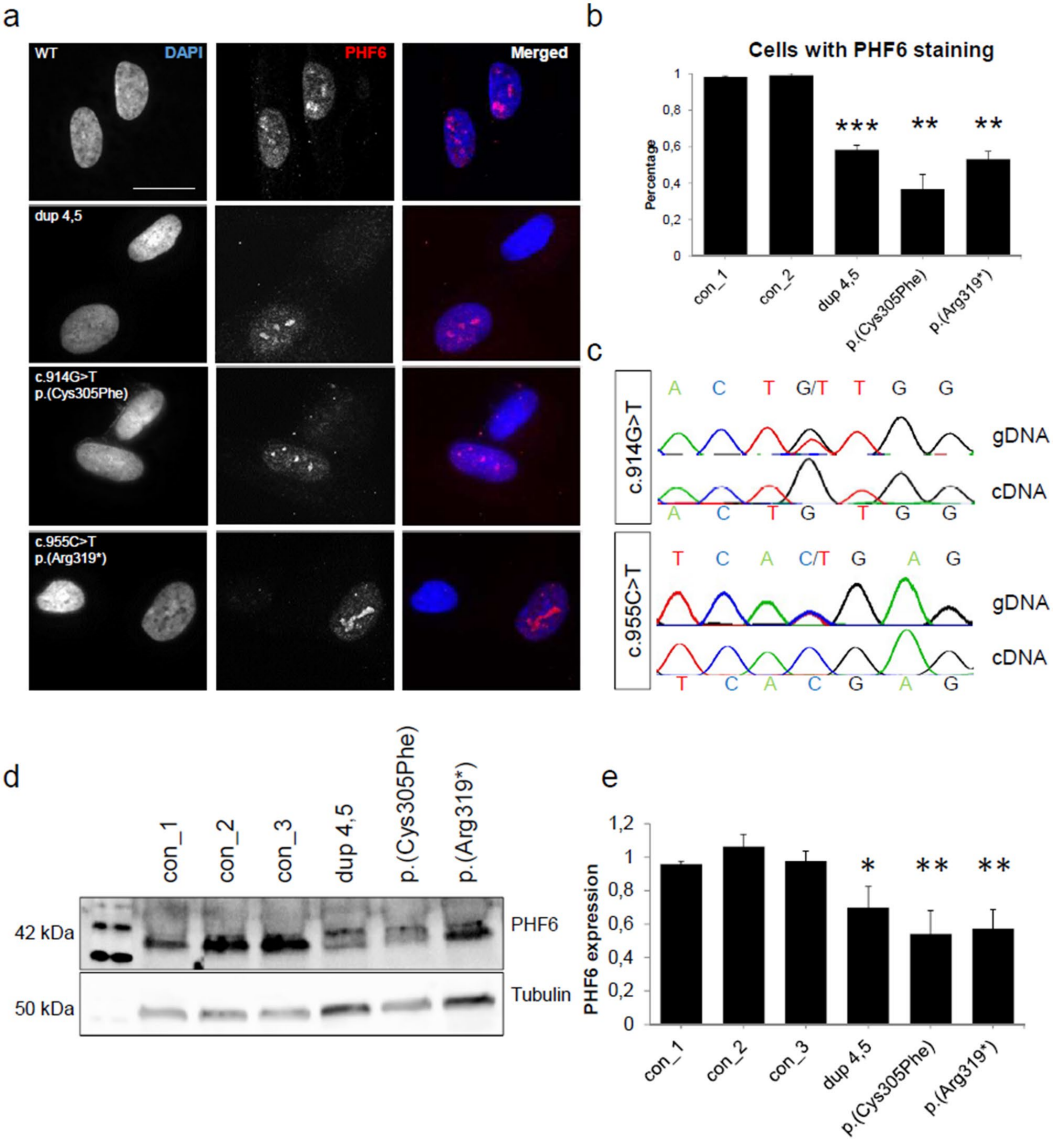
Variants in *PHF6* in female individuals lead to a loss of protein. In fibroblasts of female individuals with variants in *PHF6*, only the wildtype *PHF6* allele was expressed. (**a**) Immunofluorescence of fibroblasts of three individuals with either a duplication of exons 4 and 5, a missense variant c.914G>T (p.(Cys305Phe), or a truncating variant c.955C>T (p.(Arg319*) and one control individual. In the control, PHF6 expression could be observed in all fibroblast cells. In affected individuals, expression of PHF6 was present in only 50% of cells. Scale bar represents 20 µm. Cells were stained with rabbit polyclonal anti-PHF6 antibody (HPA001023, Sigma-Aldrich, 1:50). (**b**) Numerical analysis of fibroblasts with variants in *PHF6* and controls. Affected individuals showed expression of PHF6 in 30–50% of cells, respectively. For each individual, at least 63 cells were evaluated. (**c**) Sanger sequencing of genomic DNA (gDNA) and of cDNA from RNA of affected individuals. In the individuals with a missense or a truncating variant respectively, both wildtype and mutant allele could be observed on gDNA level. On cDNA level, only the wildtype allele was present, thus indicating nonsense mediated mRNA decay. (**d**) Exemplary western blot from fibroblasts of control and affected individuals stained for PHF6 (Santa Cruz Biotechnology, sc-365237, 1:500) and Tubulin (ab7291, abcam, 1:10,000) as a housekeeping gene. PHF6 levels in patient fibroblasts were decreased. Grouped blots were cropped from different blots. Uncropped blots can be found in Supplementary Fig. S2. (**e**) Graphical analysis of three western blot replicates indicating 50%-70% of remaining PHF6 protein in patient fibroblasts. Error bars depict the standard deviation. Asterisks indicate statistical significance (*p < 0.05, **p < 0.01, ***p < 0.001).

Fibroblast cultures were available from three previously published female individuals with de novo variants in *PHF6* [duplication of exons 4 and 5; missense variant c.914G>T (p.(Cys305Phe)); truncating variant c.955C>T (p.(Arg319*))]^4^. Fibroblasts of male individuals with BFLS were not available for this study. As the X-inactivation in fibroblasts had been random in the investigated females (in contrast to skewed X-inactivation in blood)^4^, we were able to assess both the mutant and wildtype *PHF6* allele. Utilizing immunofluorescence, western blot and Sanger sequencing, we found that only wildtype gene and protein were expressed in fibroblasts of the three individuals (Fig. 2). Fibroblasts of affected individuals lacked PHF6 expression in 40–60% of a minimum of 60 assessed cells, respectively (Fig. 2a,b). Sequencing genomic DNA and cDNA from fibroblasts of individuals with the missense and truncating variants revealed that only the wildtype and not the mutant allele was present on RNA level, thus indicating nonsense-mediated mRNA decay (NMD) for the truncating variant (Fig. 2c). As the duplication of exons 4 and 5, if in tandem, is predicted to result in frameshifting, a truncating effect with nonsense-mediated mRNA is likely as well. As NMD is less common for missense variants^31^, we also considered other mechanisms of mRNA degradation such as micro RNA (miRNA) mediated decay^32^. Indeed, variant c.914G>T was predicted to create two novel miRNA binding sites at this locus (Supplementary Fig. S2), providing a potential explanation for the loss of the mutant allele on RNA level. Nevertheless, the exact mechanism resulting in reduced expression of PHF6 carrying the missense variant remains currently elusive.

In accordance with mRNA expression levels, western blot analysis on fibroblasts showed a reduction of protein levels to 50–70% in all three affected individuals, respectively. Western blots were normalized to tubulin levels (Fig. 2d,e, Supplementary Fig. S3). In contrast to aggregation of mutant PHF6 in vitro, all three tested *PHF6* variants ex vivo appeared to result in loss of protein, independent from their type (missense, truncating, intra-genic duplication), indicating a common loss-of-function mechanism in females with BFLS.

### Broad deregulation of genes in *PHF6* knockout cells

**Figure 3:**
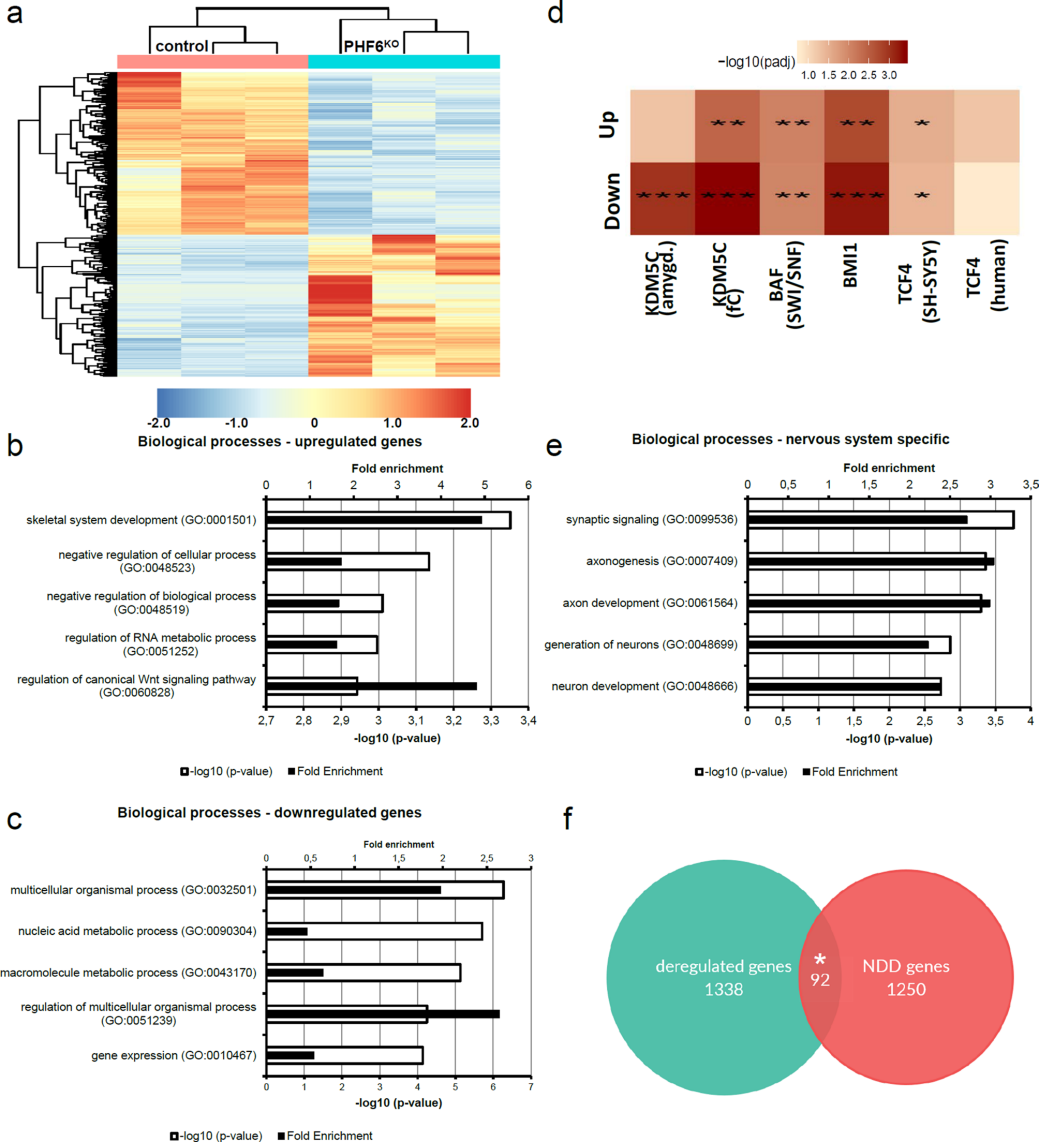
Transcriptome analysis of neuron-like *PHF6* KO cells. (**a**) Heatmap displaying 1,338 deregulated genes between differentiated SK-N-BE (2) *PHF6* KO and control cells with adjusted p value < 0.05 (FDR corrected). 626 genes were upregulated, and 712 were downregulated in *PHF6* KO cells. Heatmap was created using the pheatmap package v.1.0.12^86^ applying the standard settings (complete linkage method for hierarchical clustering for both columns and rows, scaling for rows). Blue = downregulated; red = upregulated. (**b**,**c**) Gene Ontology (GO) term analysis of (**b**) upregulated and (**c)** downregulated genes depicting the top five GO terms for biological processes. (**d**) Comparison of deregulated genes after KO/KD of common chromatin remodelers and transcription factors with deregulated genes after *PHF6* KO divided in up- and downregulated genes. Deregulated genes after KO/KD of either *KDM5C* (*Kdm5c* KO mouse model, RNA sequencing of amygdala and frontal cortex)^36^, the BAF complex (dKO *Baf155/Baf170* mouse model, RNA sequencing of the pallium of E17.5 embryos)^37^, *BMI1* (*Bmi1* KO mouse model, RNA sequencing of whole brain)^38^ or *TCF4* (*TCF4* KD SH-SY5Y neuroblastoma cells)^39^ expressed a highly significant overlap with up- and downregulated genes after *PHF6* KO. Darkness of color correlates with significance. (**e**) Significant GO terms involved in neuron development and axonogenesis (p < 0.005). (**f**) Venn diagram depicting that deregulated genes were significantly enriched for genes implicated in neurodevelopmental disorders (NDDs). Asterisks indicate statistical significance (*p < 0.05, **p < 0.01, ***p < 0.001).

Based on the observation of PHF6 loss in fibroblasts from affected female individuals, we considered knockout (KO) of *PHF6* a more suitable model for the female form of BFLS in a neuronal context than overexpression of the mutant protein. Utilizing CRISPR/Cas9 we generated *PHF6* KO in neuroblastoma SK-N-BE (2) cells. KO was confirmed by sequencing and western blot analysis in three different cell lines (Supplementary Fig. S4). Subsequently, these cells were differentiated into neuron-like cells on which we performed bulk RNA sequencing. Transcriptome analysis in three independent controls and three independent *PHF6* KO lines (targeted either exon 2 or 9, Supplementary Fig. S5) revealed a broad deregulation with 1,338 differentially expressed genes. 626 of those were upregulated, and 712 were downregulated with an adjusted p value < 0.05 (FDR corrected) (Supplementary Table S1). Controls and KO cells clustered readily within their group (Fig. 3a, Supplementary Fig. S5). The top five terms of Gene Ontology (GO) term analysis^33–35^ in upregulated genes included skeletal system development, negative regulation of cellular or biological processes, regulation of RNA metabolic processes, and regulation of canonical Wnt-signaling (Fig. 3b). Downregulated genes were enriched for genes playing a role in (regulation of) multicellular organismal processes, nucleic acid metabolic processes, macromolecule metabolic processes and gene expression (Fig. 3c). Additionally, deregulated genes were significantly (adjusted p value < 0.01) enriched for GO terms such as DNA-templated transcription and chromosome organization (Supplementary Table S1).

This prompted us to compare deregulated genes (either up- or downregulated) upon *PHF6* KO with genes deregulated after dosage alteration of a known chromatin remodeler or transcription factor. We retrieved published datasets^36–39^ containing gene expression changes caused by KO or knockdown (KD) of several genes with a clinical or possibly functional overlap to PHF6. KDM5C (MIM: 314690) (*Kdm5c* KO mouse, amygdala and frontal cortex^36^) is a demethylase which acts as a transcriptional repressor through the REST complex^40^. Variants in *KDM5C* are implicated in an X-linked NDD with mild to severe ID, epilepsy, short statue, mild facial dysmorphism including large ears, and hypogonadism (MRXSJ [MIM #300534]), partly mirroring the phenotype observed in males with BFLS^41–44^. Additionally, *KDM5C* also contains two PHD zinc finger domains^42^. The BAF complex (*Baf155/Baf170* dKO mouse, pallium of E17.5 embryos^37^) is an ATP dependent chromatin remodeling complex^45^. Variants in several subunits of the BAF complex are causative for Coffin–Siris syndrome (CSS [MIM #135900, #614607, #614608, #614609, #616938, #617808, #618362, #618779 ]), a NDD with multiple anomalies^46,47^, phenotypically overlapping with BFLS due to de novo variants in *PHF6* in young female individuals^5^. BMI1 (MIM: 164831) (*Bmi1* KO mouse, whole brain^38^) represents a major component of the polycomb group complex 1 (PcG 1), which acts as an epigenetic repressor^48,49^. As our set of deregulated genes after *PHF6* KO contained a large number of homeobox (*Hox*) genes (*HOXA1-3* [MIM: 142955, 604685, 142954]*, HOXA7* [MIM: 142950]*, HOXB2* [MIM: 142967] and *HOXC9* [MIM: 142971], Supplementary Table S1), we decided to investigate the overlap with deregulated genes after *Bmi1* KO, since the main targets of PcGs are *Hox* genes^50,51^. Lastly, we chose TCF4 (MIM: 602272) (*TCF4* KD SH-SY5Y neuroblastoma cells)^39^, a transcription factor with a major role in nervous system development^52^ and implicated in severe NDD Pitt-Hopkins syndrome (MIM: #610954). It promotes differentiation of neurons, thus being a good fit for differentiation of SK-N-BE (2) cells into neuron-like cells^53^. In addition, we used a so far unpublished list of deregulated genes in individuals with pathogenic variants in *TCF4* (patient blood cells) (Supplementary Table S1).

Deregulated genes upon *PHF6* KO were significantly overlapping with deregulated gene sets upon KO of either a Baf complex subunit or *Bmi1* (Table 1)*.* Overlapping genes between Baf complex KO and *PHF6* KO were significantly enriched for genes involved in neuron generation, development and differentiation as well as regulation of nervous system development (adjusted p value < 0.05, Supplementary Table S1). Overlapping genes between *Bmi1* KO and *PHF6* KO were enriched for genes involved in axon development, axon guidance and positive regulation of transcription by RNA polymerase II (RNAPII) (adjusted p value < 0.05, Supplementary Table S1). In addition, deregulated genes upon *PHF6* KO significantly overlapped with deregulated genes after *Kdm5c* KO and with deregulated genes after *TCF4* dosage alterations in neuroblastoma cells but not in human blood after correction for multiple testing (Table 1). Overlapping deregulated genes between *Kdm5c* KO and *PHF6* KO were enriched for synaptic signaling (adjusted p value < 0.05). Overlapping deregulated genes between *TCF4* KD and *PHF6* KO were enriched for genes involved in regulation of nervous system development, neurogenesis and cell migration (adjusted p value < 0.05). (Fig. 3d, Supplementary Table S1). This would be in line with PHF6 acting in chromatin and transcriptional regulation, especially during neuron and axon development.

**Table 1:** Overlap of deregulated genes in *PHF6* KO SK-N-BE (2) cells with other deregulated gene sets.

Overlap of PHF6 KO SK-N-BE (2) cells with	Number of downregulated genes	Adjusted p value (down)	Number of upregulated genes	Adjusted p value (up)
BAF complex^37^	30	0.009733	28	0.008548
BMI1^38^	85	0.000549	73	0.001861
KDM5C Amy^36^	27	0.001	16	0.052961
KDM5C FC^36^	34	0.000422	26	0.00385
TCF4 (SH-SY5Y)^39^	33	0.035384	32	0.029234
TCF4 (human blood)	6	0.15882	11	0.055686

Testing for statistical significance was performed using hypergeometric testing and correcting for multiple testing using the Benjamini–Hochberg procedure.

*Amy* amygdala, *FC* frontal cortex.

Furthermore, also downregulated genes after *PHF6* KO were enriched for GO terms such as neuron development, synaptic signaling, axon development and generation of neurons (Fig. 3e), suggesting a possible deregulation of neuron development contributing to the neurodevelopmental and cognitive phenotypes in BFLS. In accordance, the set of deregulated genes after *PHF6* KO was significantly (adjusted p value < 0.05) enriched for known NDD associated genes. Of 1,336 confirmed ID genes (SysID database^54^, June 2020), 1,250 were expressed in differentiated SK-N-BE (2) cells, and 92 were contained in the deregulated gene set. This is significantly higher than the expected number of 72 genes by chance (p value < 0.05) (Fig. 3f, Supplementary Table S1). Overlapping genes between the SysID data base and *PHF6* KO were enriched for genes involved in synaptic vesicle cycle, synaptic signaling, neuron projection development and generation of neurons. Genes involved in neuron projection development included *ACTB* (MIM: 102630) and *MAP1B* (MIM: 157129)*,* both important for neurite cytoskeleton^55^.

Additionally, KDM5C we compared our deregulated gene set with published lists of deregulated genes in murine cerebral cortex^56^ or agouti-related peptide (AgRP) neurons^18^ upon *PHF6* KO. We found that 18 genes were overlappingly deregulated in murine cerebral cortex and our neuron-like cells, and that 38 genes were overlappingly deregulated in murine AgRP neurons and our neuron-like cells. The overlap in both cases was significantly higher than by chance (cerebral cortex: p < 0.05, expected: 10.75; AgRP neurons: p < 0.005, expected: 23.57) (Supplementary Table S1). As, however, there was no overlap between deregulated genes from cerebral cortex and AgRP neurons, possibly due to different cell types, conclusions on specific target genes are limited.

### Impaired neurite outgrowth, proliferation and migration in *PHF6* KO cells

**Figure 4:**
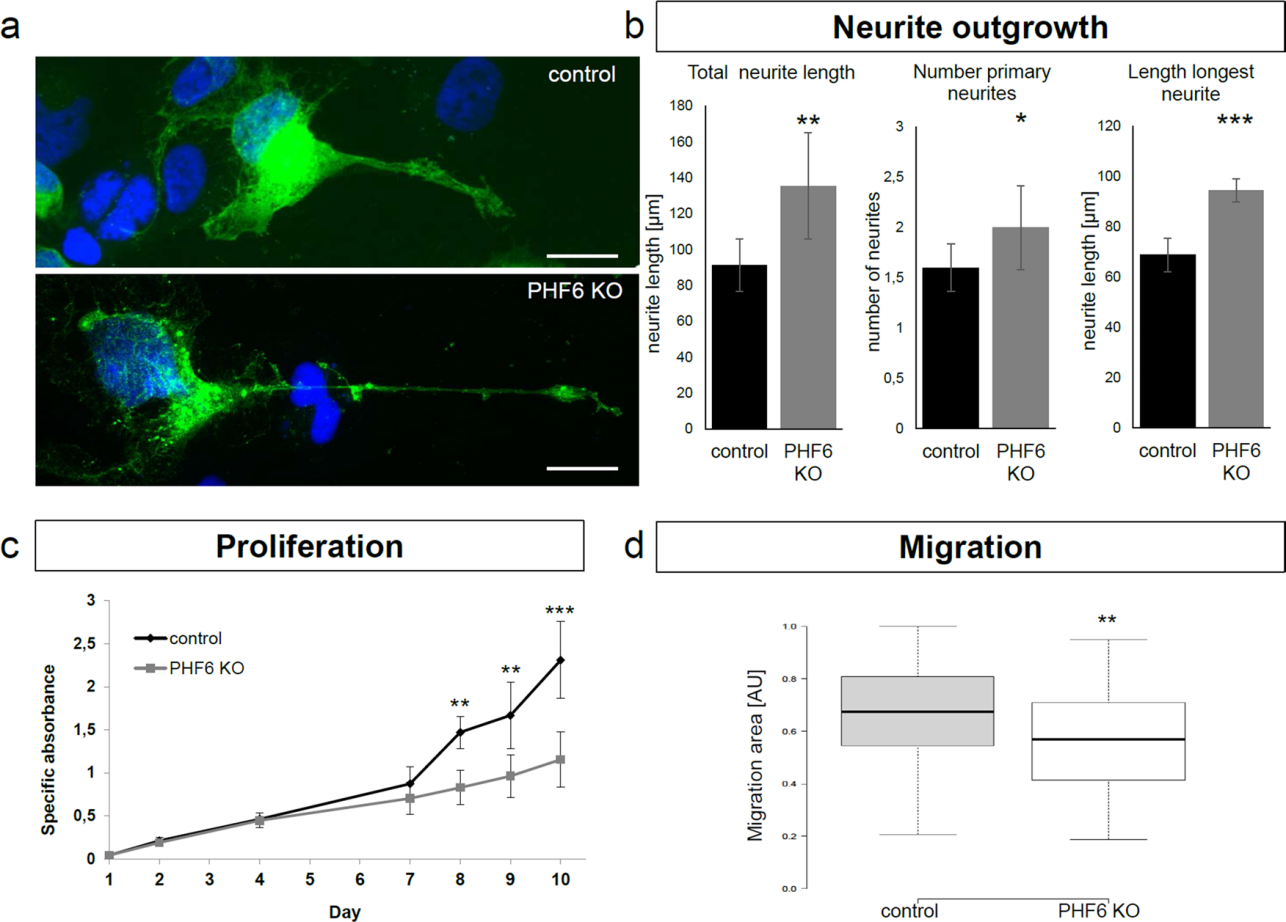
*PHF6* KO in cells reveals impaired neurite outgrowth, proliferation and migration. (**a**) Example of differentiating control and *PHF6* KO cells transfected with mCD8-GFP. The cell with *PHF6* KO had a longer primary neurite (axon) than the control cell. Scale bar depicts 20 µm. (**b**) Quantitative analysis of total neurite length, number of primary neurites and axon length (longest primary neurite) of day 10 differentiated *PHF6* KO and control cells. *PHF6* KO cells had significantly longer protrusions (total neurite length) and significantly more primary neurites. Additionally, axon length was also significantly increased in *PHF6* KO cells. Of note, while control cells developed 1–2 neurites, some of the *PHF6* KO cells had up to five neurites. For each cell line, at least 15 cells were evaluated per biological replicate. (**c**) After 8 days of differentiation, *PHF6* KO cells showed significantly impaired proliferation in comparison to control cells. (**d**) Scratch assay showing that undifferentiated *PHF6* KO cells migrated significantly slower than control cells. All experiments were performed in biological triplicates, except for the proliferation assay, which was performed in biological duplicates. Error bars depict the standard deviation. Asterisks indicate statistical significance (*p < 0.05, **p < 0.01, ***p < 0.001).

Following up on enriched GO terms such as axon development, we assessed differentiating *PHF6* KO and WT SK-N-BE (2) cells for neurite outgrowth by measuring the total length of their protrusions and length and number of primary and secondary neurites. Cells with *PHF6* KO had significantly (p value < 0.01) increased total neurite length compared to control cells (Fig. 4a,b), and the number of primary neurites was significantly increased (p value < 0.05) (Fig. 4b). Length of the longest primary neurite (axon) was also increased (p value < 0.001) (Fig. 4a,c, Supplementary Fig. S6a). While most of wild type cells rarely developed more than two primary neurites, some of the *PHF6* KO cells had up to five primary neurites (Supplementary Fig. S6b). The number and length of secondary neurites was not altered in *PHF6* KO cells compared to controls (Supplementary Fig. S6c).

We also assessed the effect of *PHF6* KO on cell proliferation using an XTT-assay. We did not observe a proliferation difference between KO and WT SK-N-BE (2) cells under normal culture conditions (Supplementary Fig. S7). However, during differentiation when kept in the presence of retinoic acid and caffeic acid, *PHF6* KO cells exhibited a significant slower proliferation starting at day eight (p value < 0.01 on day 8, p value < 0.001 on day 10) (Fig. 4c). Firstly, we explored if that might be due to cell cycle changes. We performed FACS (fluorescence activated cell sorting) experiments to determine the percentage of cells in G1, S or G2/M phase, respectively. This did not reveal any significant changes in distribution between cell cycle phases (Supplementary Figs. S8a, S9). Next, we investigated if *PHF6* KO cells might show an increase in differentiation. We performed FACS staining of cells with Ki67, a marker for cells which are not in the G0 phase^57^. Neither control nor *PHF6* KO cells were negative for Ki67 staining (< 2%), indicating that only a minor fraction of the cells were fully differentiated (Supplementary Figs. S8b, S9). Furthermore, we determined the number of DCX positive cells, a marker expressed by neuronal precursor cells and immature neurons^58^. More than 97% of control and KO cells were DCX positive, indicating unaltered differentiation (Supplementary Figs. S8c, S9). Lastly, we assessed if the number of apoptotic or dead cells might be increased in KO by using FACS staining with Annexin V and SYTOX Green Nucleic Acid Stain. We did not detect any changes in these regards (Supplementary Figs. S8d, S9). Therefore, the reason for slower proliferation of differentiating *PHF6* KO cells remained elusive.

We also assessed migration of SK-N-BE (2) cells under normal culture conditions using a wound healing assay. *PHF6* KO cells migrated significantly slower and filled a significantly smaller area of the wound after 24 h than control cells (p value < 0.01) (Fig. 4d).

These observations support a possible role of PHF6 in proper neurite outgrowth and neuron development, which might be altered in BFLS.

## Discussion

### Variant consequences on protein localisation and structure

*PHF6* belongs to the growing list of X-chromosomal genes, in which variants not only cause X-chromosomal recessive NDDs in males but also severe NDDs in females when occurring de novo^4,59–62^. Factors contributing to different manifestation in males versus females might include X-inactivation, functional mosaicism due to X-inactivation, localisation and nature of variants, different functional consequences of sex specific variants, and dosage related expression effects^4,59–62^. Although the mutational spectrum for *PHF6* includes truncating and missense variants for both genders, there are no overlapping variants reported yet occurring in both X-chromosomal recessive BFLS families and de novo in females. Additionally, variants identified in females seem to represent the severe end of the mutational spectrum. There are more truncating variants than in males, and the only missense variant is located within the C-terminal ePHD, while variant localisation in males is more variable^4^. It might therefore be speculated that variants found in females might have a more severe effect than variants found in males and might be lethal when occurring hemizygously. This is supported by the fact that complete knockout of PHF6 is lethal in mice^56,63^. Such a severe effect would be in line with our observation of complete loss of mutant *PHF6* on both mRNA and/or protein level in fibroblasts of three females, independent from the variant (2-exon duplication, truncating variant, missense variant). In a previous study on variants identified in male individuals with BFLS, a hypomorphic effect was discussed^56^. This is supported by several findings. A mouse model with CRISPR/Cas9 mediated knock-in of the specific c.296G>T (p.(Cys99Phe)) variant showed unaltered mRNA expression and only reduced protein levels of mutant *PHF6*^56^. Furthermore, while the only missense variant identified in females is residing in the C-terminal ePHD, missense variants identified in males are located in either the N-terminal or the C-terminal ePHD. The N-terminal ePHD mediates the interaction of PHF6 with UBTF^12,14^, and the C-terminal ePHD interacts with double stranded DNA (dsDNA) through its positively charged region^9^. NMR spectra analysis of the four missense variants of male origin in the C-terminal ePHD did not reveal a structural impairment, while dsDNA binding abilities were still almost completely abolished^9^. Whereas structural modelling in our study indicated a severe effect on protein structure for variants in the N-terminal ePHD, it is predicted to be less severe for male variants in the C-terminal ePHD. In contrast, the female missense variant in the C-terminal ePHD appears to have a severe effect on protein structure. This suggests that in males a more severe structural effect on the N-terminal ePHD is required to result in a comparable impairment of PHF6 function as from variants in the C-terminal ePHD. As a logic implication, a severe alteration in the important C-terminal ePHD might be lethal in males and result in a severe NDD in heterozygous females. The association of inherited variants in X-linked recessive BFLS families with no or only mild phenotypes in carrier females might therefore be due to a less severe effect on protein function. However, we cannot exclude that variants in *PHF6* occurring in male individuals also result in complete loss of protein, especially those with high destabilizing effects on the N-terminal ePHD.

Though it is debatable—in light of the observations from fibroblasts—if overexpression of mutant *PHF6* is a suitable model for BFLS, it is interesting to see that there are also differences in the subcellular localisation and protein levels depending on the variant occurrence in males or females.

Therefore, in addition to X-inactivation related functional mosaicism, also more severe functional consequences of pathogenic *PHF6* variants in females might contribute to their severe and distinct phenotype. This suggests a possible genotype–phenotype correlation regarding gender-based localisation and severity of *PHF6* variants.

### PHF6 is involved in chromatin/transcriptional regulation

There are several lines of evidence pointing to PHF6 acting as a chromatin/transcription regulator, among them its domain structure^2,9^, its nuclear localisation^8^ and co-localisation with euchromatin^8^, and its interaction with several chromatin remodelling or transcription regulating proteins and complexes^12,13,16,64^. Two recent studies used ChIP (chromatin immunoprecipitation) sequencing to show that PHF6 binds chromatin similar to typical chromatin remodelers^17,18^. Furthermore, transcriptional deregulation as a consequence of *Phf6* knockdown or knockout was demonstrated in the nervous system of two independent mouse models^17,56^ and one rat model^12^. Using a human, neuron-like cell line in our study, we also found a broad deregulation of genes upon KO of *PHF6*. Deregulated genes significantly overlapped with deregulated genes after dosage alterations of known chromatin remodelers or transcription factors such as KDM5C, the BAF complex, BMI1 and TCF4, suggesting that PHF6 might act in a similar way. Interestingly, all overlapping genes were enriched for genes involved in either neuron generation and differentiation, or even more specific in axon development and guidance and synaptic signalling, proposing a specific role for PHF6 in these processes. In detail, the highest overlap was found with deregulated genes after either *Kdm5c* KO in the frontal cortex or *Bmi1* KO. Pathogenic variants in *KDM5C* are associated with a NDD, partly mirroring the phenotype observed in males with BFLS^42–44^. Therefore, commonly deregulated genes, most prominently involved in synaptic signalling, might contribute to these similarities. BMI1 is a major component of the polycomb group complex 1 (PRC1), which is an essential epigenetic repressor of multiple regulatory genes involved in embryonic development^48–50,65^. This is also in line with a recent study identifying PHF6 as a transcriptional repressor of activity-dependent immediate-early genes^18^ and other studies discussing repressing effects^9,17^. However, repressing function might only apply for specific pathways, as deregulated genes both in our as well as other transcriptome studies^12,17,18,56^ indicated both upregulating and downregulating capacities. Furthermore, Bmi1 is required for the repression of *Hox* genes, preserving the undifferentiated state of stem cells and preventing inappropriate differentiation^50^. We also observed an upregulation of *Hox* genes after *PHF6* KO, suggesting a potential similar mechanism. Interestingly, deregulated genes upon *PHF6* KO also significantly overlapped with deregulated genes after KO of BAF (SWI/SNF) complex subunits in mouse (pallium of E17.5 embryos). Variants in multiple subunits of the BAF are causative for Coffin–Siris syndrome, a NDD with multiple anomalies^46,47^. As phenotypic overlap between BFLS in females and CSS has been acknowledged before^5,66,67^, commonly deregulated genes, especially enriched for genes involved in neuron generation, development and differentiation, might contribute to the phenotypic similarities between these disorders.

### PHF6 is involved in neuron development

Deregulated genes in our study were mainly enriched for genes involved in broad processes such as transcriptional and chromatin regulation, but also for more specific processes such as axon and neuron development. Following-up this observation in neuron-like cells, we indeed observed a significantly increased total length of protrusions, an increased number of primary neurites and increased axon length. Altered neurite development has been demonstrated in several other NDD models before. For example, shRNA mediated knockdown of *Kiaa2022* (*KIAA2022* [MIM: 300524]), which is implicated in an X-chromosomal NDD in both males and females (MRX98 [MIM: #300912]), in mice resulted in longer or shortened apical neurites dependent on the developmental timepoint^68^. Also shRNA mediated knockdown of *Ankrd11* (*ANKRD11* [MIM: 611192]), of which haploinsufficiency in humans is associated with neurodevelopmental KBG syndrome (MIM: #148050), resulted in an increased number of shortened neurites in a mouse model^69^. Apart from neurite development also aberrant migration and differentiation of neurons and precursor cells can be observed in NDDs^69–73^. During radial migration, pyramidal neurons undergo a transition from multipolar to bipolar morphology in the intermediate zone (reviewed in ^74^). Impairment of this transition process in cortical pyramidal neurons and subsequently radial migration defects were described for the *Ankrd11* mouse model^69^. In accordance, we observed significantly slower migration of SK-N-BE (2) cells after *PHF6* KO compared to controls. In combination with the observation of an increased number of neurites this might be in line with a disturbed transition from multi- to bipolar neurons. Impaired migration has been discussed in the context with BFLS before^12^. An RNAi based knockdown of *Phf6* in the developing mouse cerebral cortex at E14 showed impaired migration of cortical neurons as well as an increased number of multipolar neurons and a concomitantly reduced number of bipolar neurons^12^. However, in another mouse model carrying a patient specific *Phf6* variant (p.Cys99Phe), no migration defects were reported^56^. Of note, there is also a report on two adult female individuals with the identical de novo duplication of exons 4 and 5 in *PHF6* and presenting with seizures, in whom MRI anomalies were described that would be compatible with malformations of cortical development based on abnormal neuronal migration^75^. Furthermore, PHF6 is directly targeted and suppressed by miR-128 in the developing cortex. Their interaction is critical for proper migration and dendritic outgrowth of upper layer neurons^76^.

Apart from altered neurite outgrowth and migration we also observed altered proliferation in our cell model, another anomaly often observed in NDDs^73^. Differentiating SK-N-BE (2) cells after *PHF6* KO proliferated significantly slower than control cells, while SK-N-BE (2) cells under normal cell culture conditions did not exhibit changes Previous studies showed that stable knockdown of *PHF6* in HeLa cells under normal cell culture conditions resulted in decreased cell proliferation starting on day five^14^, while CRISPR/Cas9 mediated *Phf6* KO in B-ALL cells did not show any proliferation changes^17^, and *Phf6* KO in hematopoietic stem cells resulted in better growth^77^. This might indicate that the effect of PHF6 loss on proliferation might be cell type specific. For differentiating SK-N-BE (2) cells, we investigated if underlying mechanisms might be apoptosis, cell death, accelerated differentiation or cell cycle changes. A role of PHF6 in cell cycle regulation has been controversially discussed^14,15,17,19^. We could not detect changes in cell cycle regulation or any of these other processes, leaving the reason for the proliferation defect unclear.

Taken together, our observations suggest that PHF6 might act as a context-specific epigenetic activator/repressor and is required for correct migration, proliferation and development of neurons. Also dependent on the severity of variants (males versus females), impairment of these processes might contribute to the neurodevelopmental and cognitive dysfunction in BFLS.

## Material and methods

### Structural modelling

The structural effects of variants located in the C-terminal ePHD were assessed based on the crystal structure of this domain (PDB code: 4NN2^9^). This structure also served as a template to model the N-terminal ePHD, which exhibits 47% sequence identity to the C-terminal ePHD. Modelling was performed with HHpred^78^ and Modeller^79^. The structural effect of the variants was assessed using VIPUR^30^, which is designed to distinguish between neutral and deleterious protein variants by modelling their effect on the three-dimensional protein structure.

### Cell lines

This study was approved by the ethical review board of the University Erlangen-Nuremberg, and informed consent was obtained from healthy control individuals, parents (underaged participants) or legal guardians (affected adult participants). The work described here was conducted in accordance with relevant guidelines and regulations.

Skin fibroblasts were obtained from three previously published female individuals^4^ with de novo variants in *PHF6.* HEK-293, SK-N-BE (2) cells and fibroblasts were cultured at 37 °C and 5% CO_2_ under sterile conditions. For differentiation of neuroblastoma SK-N-BE (2) cells into neuron-like cells, they were seeded into 6-well plates and differentiated for 10 days using 10 µM all-trans retinoic acid and 25 µM caffeic acid. Medium was changed every 2–3 days (protocol was adapted from^80^).

### Protein, RNA and DNA analysis

Human *PHF6* was amplified from cDNA derived from whole blood and cloned into a pCR 2.1-TOPO vector (Thermo Fisher Scientific). After site-directed mutagenesis using a modified version of the QuikChange Site-Directed Mutagenesis protocol (Stratagene)*,* wildtype and mutant *PHF6* cDNAs were transferred into an N-terminal HA-tagged CMV expression vector. C-terminal myc-tagged UBE3A in a pCMV3 expression vector was used as transfection control.

For immunofluorescence experiments, cells were grown on poly-lysine coated coverslips and transiently transfected with the plasmids using jetPrime (Polyplus-transfection) following manufacturer’s instructions. Cells were fixated with 4% paraformaldehyde in PBS for 10 min at room temperature (RT) or with MetOH + EGTA for 10 min at − 20 °C 24 or 48 h post transfection. Cells were stained with rabbit polyclonal anti-PHF6 antibody (HPA001023, Sigma-Aldrich, 1:50), or rabbit anti-HA (H6908, Sigma-Aldrich, 1:75) and mouse monoclonal anti-Nucleolin antibody (39-6400, Thermo Fisher Scientific, 1:200), and with Alexa Fluor 488 goat anti-mouse (A11001, Thermo Fisher Scientific, 1:1,500), and/or Alexa Fluor 546 donkey anti-rabbit (A10040, Thermo Fisher Scientific, 1:1,500), and DAPI (SERVA, 1:25,000) for nuclei counterstaining. Cells were analyzed with a Zeiss Axio Imager Z2 Apotome microscope with a 63 × objective. For analysis of patient fibroblasts, at least 63 cells per cell line were analyzed, and the number of PHF6 expressing cells was counted. For analysis of HA-tagged mutant and wildtype PHF6, at least 40 cells per construct were analyzed.

RNA was extracted from cells using the RNeasy Mini Kit (QIAGEN) following manufacturer’s instructions. DNase digestion was performed on-column with the RNase-free DNase kit (QIAGEN). Reverse transcription of RNA into cDNA was performed using the SuperScript II reverse transcriptase (Thermo Fisher Scientific).

Genomic DNA (gDNA) was extracted from cells using the DNeasy Blood & Tissue Kit (QIAGEN) following manufacturer’s instructions.

For analysis of patient variants in *PHF6* on RNA and gDNA level, Exon 9 was amplified (primer sequences exon 9: F: tagatgtcagacctgggatgg; R: ttggatggatgaatgctaatc) and sequenced.

For protein analysis, cells were lysed in Lysis Puffer (0.01 M Tris, 0.15 M NaCl, 1%Triton-X, 1%Protease Inhibitors, pH 7.5) and frozen for 1 h at − 80 °C.

For western immunoblotting, the Mini PROTEAN TGX stain free electrophorese system (Bio-Rad, 1658005) in combination with the Trans-Blot Turbo Transfer System (Bio-Rad, 1704150) was used. After blocking, membrane was incubated with either rabbit polyclonal anti-PHF6 (HPA001023, Sigma-Aldrich, 1:200), mouse monoclonal anti-PHF6 (sc-365237, Santa Cruz Biotechnology, 1:500), mouse anti-tubulin (ab7291, abcam, 1:10,000), mouse anti-myc (M4439, Sigma-Aldrich, 1:5,000) or rabbit anti-HA (H6908, Sigma-Aldrich, 1:500) antibody for 1.5 h at RT or overnight at 4 °C. Whole protein or alpha-tubulin were used as loading controls. C-terminal myc-tagged UBE3A was used as transfection control. Secondary, HRP-conjugated antibody (goat anti-rabbit, 170-6515, Bio-Rad, 1:10,000; or goat anti-mouse, ab97023, abcam, 1:10,000) was applied for 1 h. Blots were stained with SuperSignal West Femto Maximum Sensitivity Substrate, scanned using the ChemiDoc Imaging System (Bio-Rad, 17001401), and analyzed using the Image Lab software version 6.0.0 (Bio-Rad). Mutant HA-tagged PHF6 was normalized using transfection control UBE3A (myc-tagged).

### CRISPR/Cas9 mediated *PHF6* KO

SK-N-BE (2) cells were subjected to CRISPR/Cas9 mediated KO using the GeneArt CRISPR Nuclease Vector Kit (Life Technologies). *PHF6* specific crRNA sequences were created using the CRISPR Targets Track from UCSC Genome Browser on Human Feb. 2009 (GRCh37/hg19) Assembly. Two crRNAs were designed, targeting exon 2 or 9, respectively. The guide sequence, efficiency as well as potential off targets can be found in Supplementary Table S1 and S2. For completion of the GeneArt CRISPR Nuclease Vector, the crRNA was cloned into the vector following manufacturer’s instructions. Knockout was performed following manufacturer’s instructions. To achieve transfected single cell colonies, after 24 h transfected cells were resuspended in 1 × PBS supplemented with 2% FCS and 2 mM EDTA and sorted into 96-wells using FACS and OFP as a fluorescent marker. Single cell colonies were grown until confluency and transferred into one well of a six well plate. Genomic DNA was extracted and sequenced to assess for gene disrupting variants (Supplementary Fig. S4a). Positive clones were validated using western blotting (Supplementary Fig. S4b–d).

### RNA sequencing and transcriptome analysis

RNA sequencing was executed in-house at the NGS core unit of the Medical Faculty of the FAU Erlangen. The TruSeq Stranded mRNA LT Sample Prep Kit (illumina, San Diego, CA) was used for library preparation. Libraries were subjected to single-end sequencing (101 bp) on a HiSeq-2500 platform (illumina, San Diego, CA, USA). The obtained reads were converted to .fastq format and demultiplexed using bcl2fastq v2.17.1.14. Quality filtering was performed using cutadapt v. 1.15^81^; then reads were mapped against the human reference genome (Ensembl GRCh37, release 87) using the STAR aligner v. 2.5.4a^82^, and a STAR genome directory created by supplying the Ensembl gtf annotation file (release 87). Read counts per gene were obtained using featureCounts program v. 1.6.1^83^ and the Ensembl gtf annotation file.

Following analyses were performed using R version 3.5.0^84^. In particular, differential expression analysis and principal component analysis (PCA) was performed with the DESeq2 package v.1.20.0^85^. Heatmap was created using the pheatmap package v.1.0.12 applying the standard settings (complete linkage method for hierarchical clustering for both columns and rows, scaling for rows)^86^.

Enrichment analysis was performed using four already published data sets^36–39^ and one in house so far unpublished list of deregulated genes after TCF4 dosage alterations from RNA sequencing and transcriptome analysis of RNA from peripheral blood, collected and extracted with the PaxGene system (PreAnalytiX, BD and QIAGEN, Hombrechtikon, Switzerland) from three individuals with variants in *TCF4* (Supplementary Table S1). RNA sequencing was performed as described above. The KDM5C deregulated gene dataset was retrieved from a *Kdm5c* KO mouse model, where the authors dissected the amygdala and the frontal cortex from three adult mice for each genotype (KO/WT) and performed RNA sequencing^36^. The BAF-complex deregulated gene dataset was retrieved from a conditional dKO *Baf155/Baf170* mouse model where both genes where knocked out in late cortical neurogenesis. RNA was extracted from the pallium of E17.5 embryos and RNA sequencing was performed^37^. The BMI1 deregulated gene dataset was retrieved from adult brain tissue from a *Bmi1* KO mouse model^38^. The TCF4 deregulated gene dataset was retrieved from *TCF4* KD SH-SY5Y neuroblastoma cells^39^. Firstly, deregulated genes in mice were converted to human gene IDs using BioMart^87^. A common background was generated by using all genes with a base mean > 2 (if applicable) that were present in both data sets. Next, deregulated genes present in shared background were computed. Lastly, overlap of these deregulated genes was computed. Lists can be found in Supplementary Table S1. Testing for statistical significance was performed using hypergeometric testing and correcting for multiple testing using the Benjamini–Hochberg procedure. GO term analysis was performed on overlapping genes.

For comparison with confirmed ID genes (SysID database^54^, June 2020), the number of confirmed ID genes present in SK-N-BE (2) transcriptome data was analyzed and the overlap with deregulated genes was computed. Statistical testing was performed using hypergeometric testing. GO term analysis was performed on overlapping genes.

Deregulated genes were compared with RNA sequencing datasets from two different studies^18,56^. Data was processed as described above for enrichment analysis.

### Measurement of neurites

For neurite analysis SK-N-BE (2) cells were transfected with 1 µg of mCD8-GFP using jetPRIME (Polyplus-transfection) following manufacturer’s instructions on day eight of differentiation. 24 h post transfection medium was changed. Cells were subjected to immunofluorescence analysis at differentiation day 10 as described above using only DAPI as counterstaining.

Cells were analyzed with a Zeiss Axio Imager Z2 Apotome microscope. Primary and secondary neurite length was measured using the NeuronJ plugin^88^ in Fiji^89^ and respective number was counted. Three *PHF6* KO and three control cell lines were evaluated, for each line at least 30 cells were measured. The experiment was performed in biological triplicates. Data was tested for normality using the Kolmogorov–Smirnov test. Testing for statistical significance was performed using a two-tailed t test.

### XTT assay

Proliferation of undifferentiated and differentiating cells was assessed using the CyQUANT XTT Cell Viability Assay (invitrogen). Three *PHF6* KO and three control cell lines were seeded in 96-well plates and either kept in normal medium for 4 days or differentiated for 10 days. Every day, four wells per cell line were treated with the XTT reagent following manufacturer’s instructions and incubated for 4 h. The color change was measured using a plate reader at wave length 660 nm and 450 nm. The specific absorbance of each well was determined using following formula: Specific Absorbance = [Abs450 nm(Test) −Abs450 nm(Blank)] −Abs660 nm(Test). The experiment was performed in biological duplicates and technical quadruplicates. Data was tested for normality using the Kolmogorov–Smirnov test. Testing for statistical significance was performed using a two-tailed t test.

### Wound healing assay

Migration of *PHF6* KO and control cells was assessed using four well culture inserts (ibidi) following manufacturer’s instructions. SK-N-BE (2) cells were seeded into each well of the culture inserts, which were removed after 24 h. Resulting cell gaps were photographed every 4 h up to 24 h. The growth area was measured using the Wound healing tool^90^ on Fiji^89^. The experiment was performed in biological triplicates using four control cell lines and eight *PHF6* KO cell lines. Data was tested for normality using the Kolmogorov–Smirnov test. Testing for statistical significance was performed using a two-tailed t test.

## Supplementary information

Supplementary Figures.

Supplementary Tables.

## Data Availability

OMIM, https://www.omim.org/. Gene Ontology, https://geneontology.org/. SysID database, https://sysid.cmbi.umcn.nl/.
