# Supplementary material for: Loss of PHF6 leads to aberrant development of human neuron-like cells
Source: Sci Rep. 2020 Nov 4;10:19030. doi: 10.1038/s41598-020-75999-2 (PMC7642390; doi:10.1038/s41598-020-75999-2)
Supplement: Supplementary file 1 — Supplementary Figures. [file 41598_2020_75999_MOESM1_ESM.pdf]

Supplementary Information

Loss of PHF6 leads to aberrant development of human neuron-like cells

Anna Fliedner[1], Anne Gregor[1], Fulvia Ferrazzi[1], Arif B. Ekici[1], Heinrich Sticht[2], Christiane Zweier[1,3*]

[1]Institute of Human Genetics, Friedrich-Alexander-University Erlangen-Nürnberg, 91054 Erlangen, Germany

[2]Institute of Biochemistry, Friedrich-Alexander-Universität Erlangen-Nürnberg, 91054 Erlangen, Germany

[3]Department of Human Genetics, Inselspital, Bern University Hospital, University of Bern, 3010 Bern, Switzerland

* Correspondence to: Christiane Zweier, Department of Human Genetics, University Hospital and University Bern, Freiburgstrasse 15, 3010 Bern, Switzerland

Phone: +41 31632 9446

Email: christiane.zweier@insel.ch

Supplementary Figures

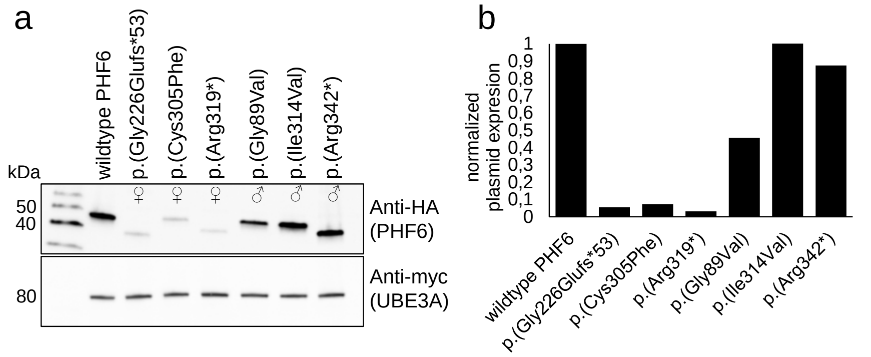

c

Plasmid	Quantification
wildtype PHF6	1
p.(Gly226Glufs*53)	0,0548323
p.(Cys305Phe)	0,0724577
p.(Arg319*)	0,0314577
p.(Gly89Val)	0,4569798
p.(Ile314Val)	1,059356
p.(Arg342*)	0,87533

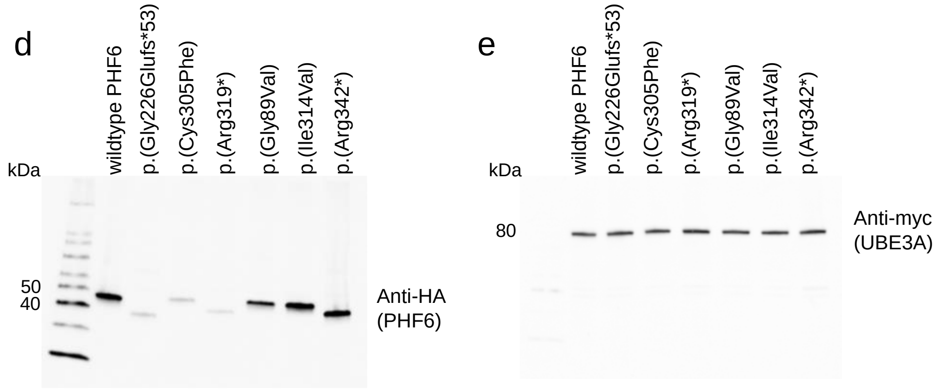

Supplementary Figure S1. Protein levels of overexpressed, mutant PHF6

HA-tagged wildtype and mutant *PHF6* containing two truncating or a missense variant identified *de novo* in three females (c.677delG, p.(Gly226Glufs*53); c.914G>T, p(Cys305Phe); c.955C>T, p.(Arg319*)), or two missense and one truncating variant occurring in males (c.266G>T, p.(Gly89Val); c.940A>G, p.(Ile314Val); c.1024C>T, p.(Arg342*)), in combination with myc-tagged UBE3A as transfection control were transiently overexpressed in HEK-293 cells. (a) Western Blot analysis of transiently transfected HEK-293 cells (0.5 µg WT or mutant HA-PHF6 and 0.5 µg UBE3A-myc). Variants occurring in male individuals showed no or slightly reduced expression, while the expression of PHF6 with variants found in females was strongly diminished. UBE3A-myc was used as transfection and normalization control. Blot was stained for HA (H6908, Sigma-Aldrich, 1:500) and myc (M4439, Sigma-Aldrich, 1:5,000). Blots were cropped from two different blots. Full length blots are shown in (d) and (e). (b, c) Quantification of expressed plasmids normalized on UBE3A-myc as transfection control. All variants in female individuals led to a drastic decrease of plasmid expression (3 - 7% remaining protein, respectively). Missense variant p.(Gly89Val) occurring in a male individual and residing in

ePHD1 led to reduced expression (46% remaining protein), while a missense variant in ePHD2 did not affect expression. A truncating variant resulted in a slight reduction (88% remaining protein). (d, e) Uncropped western blots.

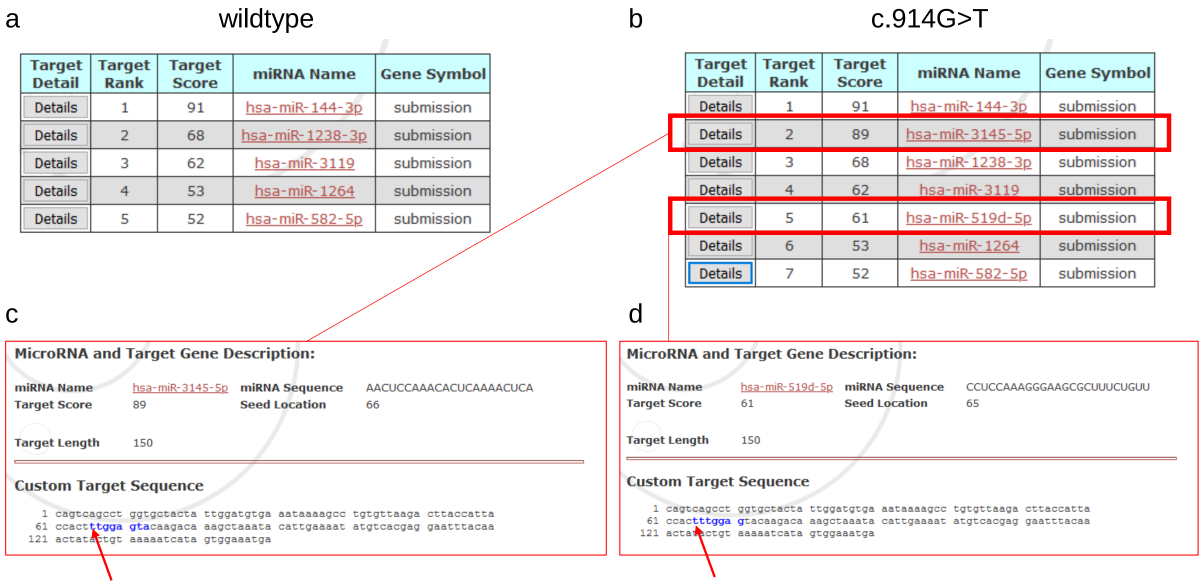

Supplementary Figure S2. Micro RNA binding sites at c.914

Micro RNA binding sites close to variant c.914G>T. (a) In wildtype *PHF6*, five miRNA binding sites were detected around position c.914G, but no miRNA was directly binding at c.914G. (b) Variant c.914G>T was predicted to create two novel miRNA binding sites at that locus, those can be observed in (c) and (d). Red arrow is marking position c.914. Micro RNA binding sites were predicted using http://mirdb.org/[1; 2].

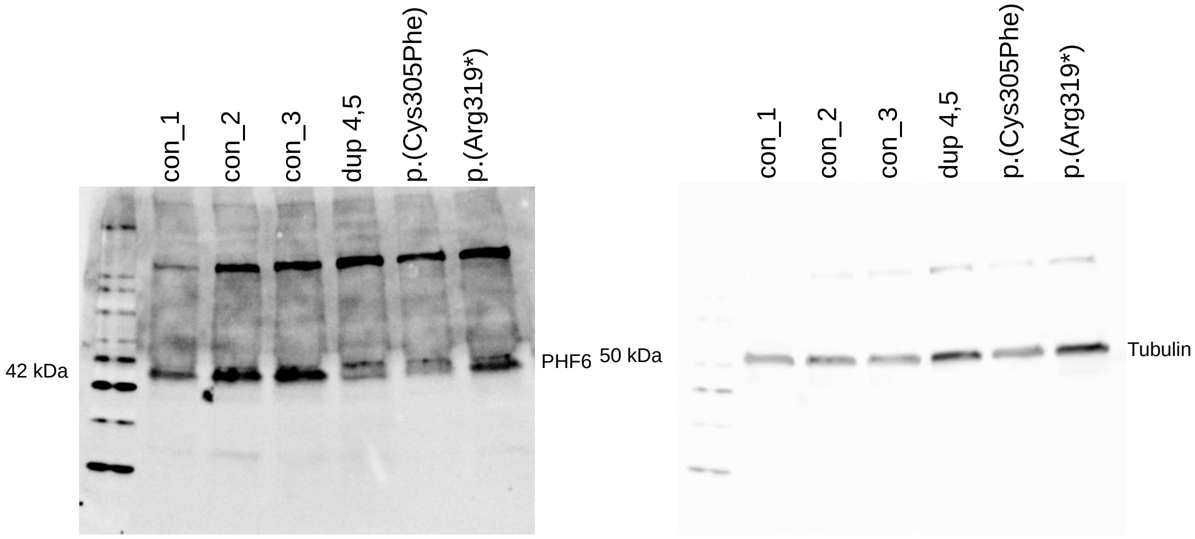

Supplementary Figure S3. Western blot of fibroblasts from affected individuals and controls

Exemplary uncropped western blots from **Fig. 2d** from control and patient fibroblasts stained for PHF6 (Santa Cruz Biotechnology, sc-365237, 1:500) and Tubulin (ab7291, abcam, 1:10,000) as a housekeeping gene.

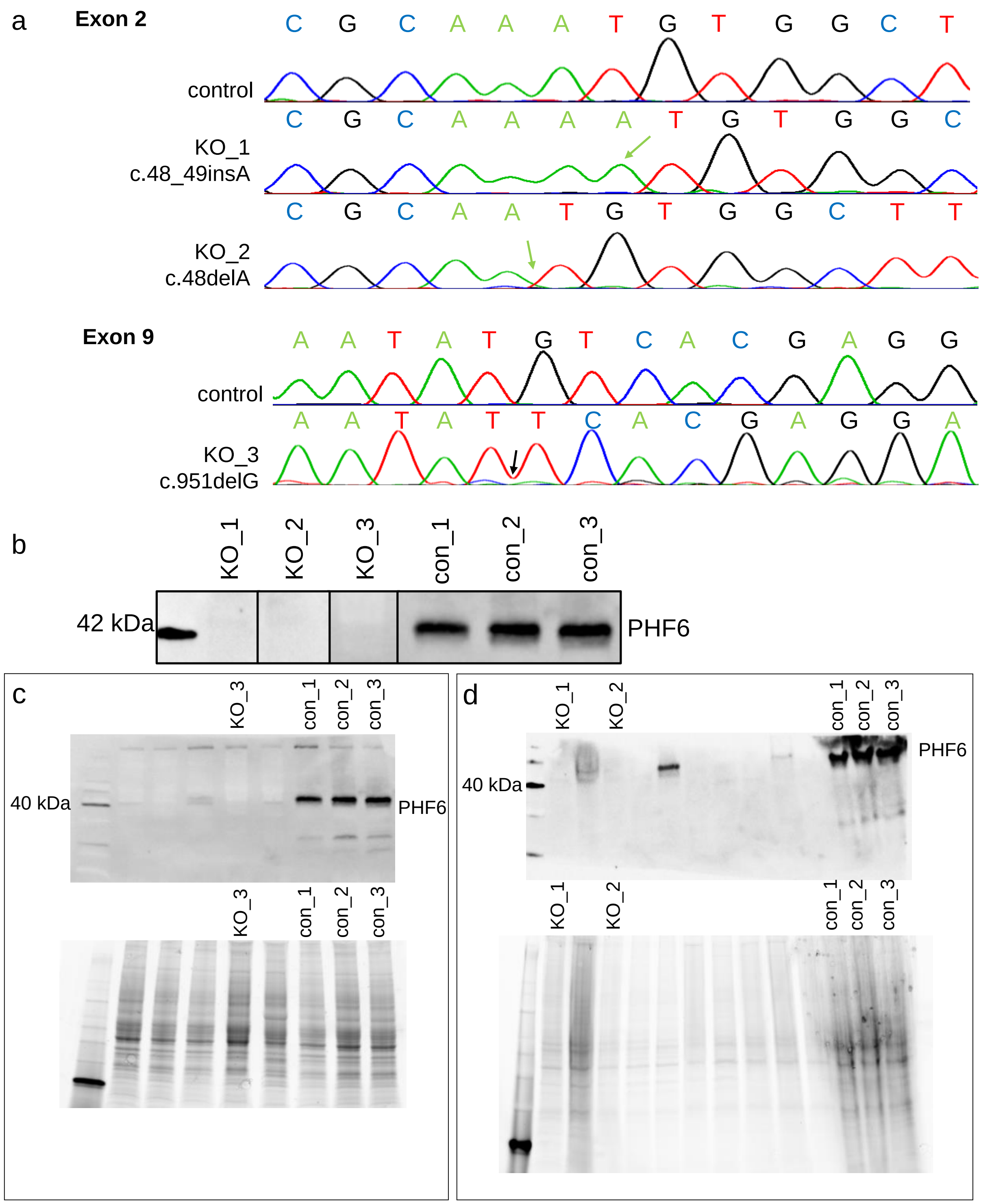

Supplementary Figure S4. Validation of CRISPR/Cas9-mediated *PHF6* knockout

(a) Genomic DNA from control and *PHF6* KO SK-N-BE (2) cell colonies was extracted and sequenced. Two gene disrupting variants in exon 2 (KO_1, c.48_49insA; KO_2, c.48delA) and one gene disrupting variant in exon 9 (KO_3, c.951delG) were chosen for further analysis. (b) Western blot of three control cell lines and three PHF6 knockout (KO) cell lines demonstrating successful KO of PHF6. Blot was stained for PHF6 (Santa Cruz Biotechnology, sc-365237,

1:500). Blots were cropped from two different blots (see **Supplementary Fig. S4c,d**). (c,d) Uncut western blots as well as whole protein blot used as loading control.

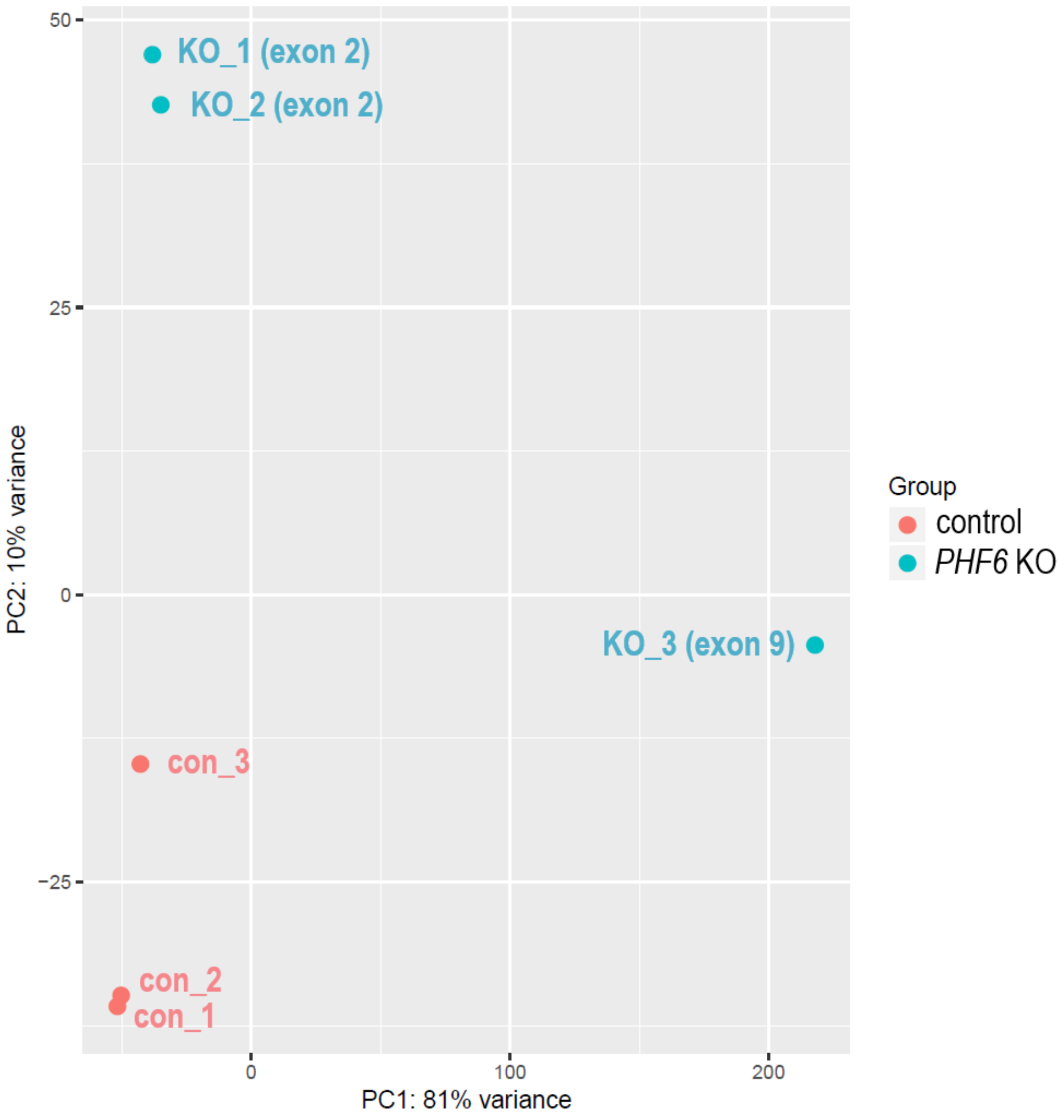

Supplementary Figure S5. Principal component analysis (PCA) of *PHF6* KO and control cells

Principal component analysis (PCA) on neuron-like cells showed clear separation between *PHF6* KO and control cells. Of note, the knockout of KO_1 and KO_2 was created by targeting exon 2, the knockout of KO_3 by targeting exon 9. This could explain the separation on PCA. PCA plot was created using the DESeq2 package version 1.20.0[3].

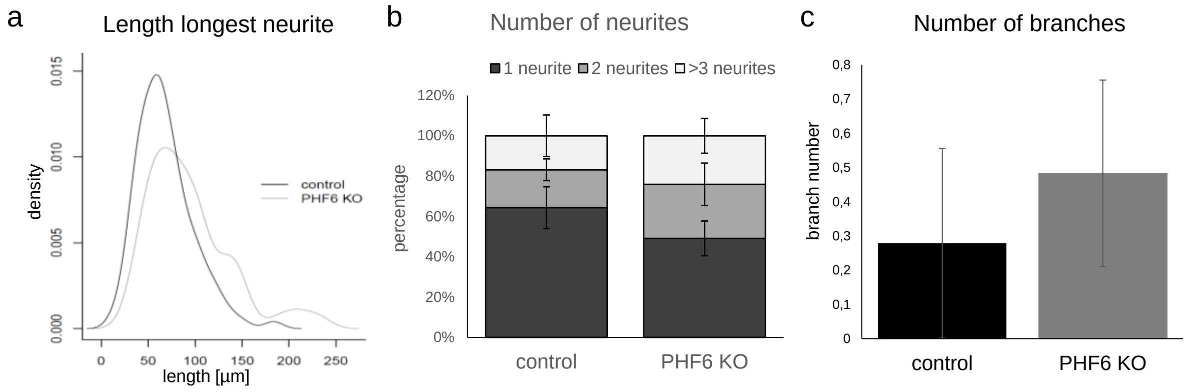

Supplementary Figure S6. Analysis of neurite length and branching of differentiated SK-N-BE (2) cells

(a) Plot depicting frequency of longest primary neurite (axon) lengths. Note a shift in the curve towards longer axon length in *PHF6* KO cells. (b) Quantitative analysis of primary neurite number of day 10 differentiated *PHF6* KO and control cells. Of note, while control cells developed 1 – 2 primary neurites, some of the *PHF6* KO cells had up to five primary neurites. (c) Quantitative analysis of the number of branches of day 10 differentiated *PHF6* KO and control cells. There was no difference in the number of branches/secondary neurites between control and KO cells.

Experiment was performed in biological triplicates with at least 15 cells per replicate. Error bars depict the standard deviation.

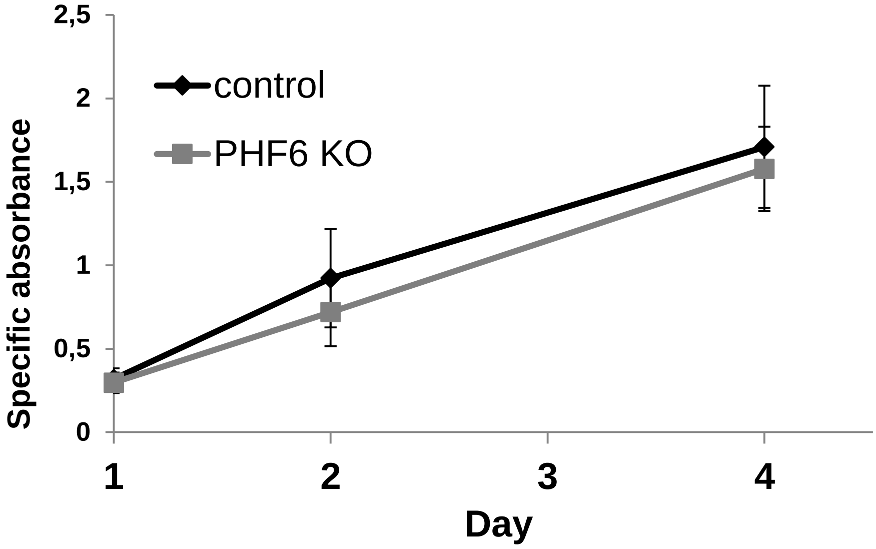

Supplementary Figure S7. Analysis of proliferation of undifferentiated SK-N-BE (2) cells

Undifferentiated *PHF6* KO and control cells showed no difference in proliferation using the XTT assay. Experiment was performed in biological duplicates. Error bars depict the standard deviation.

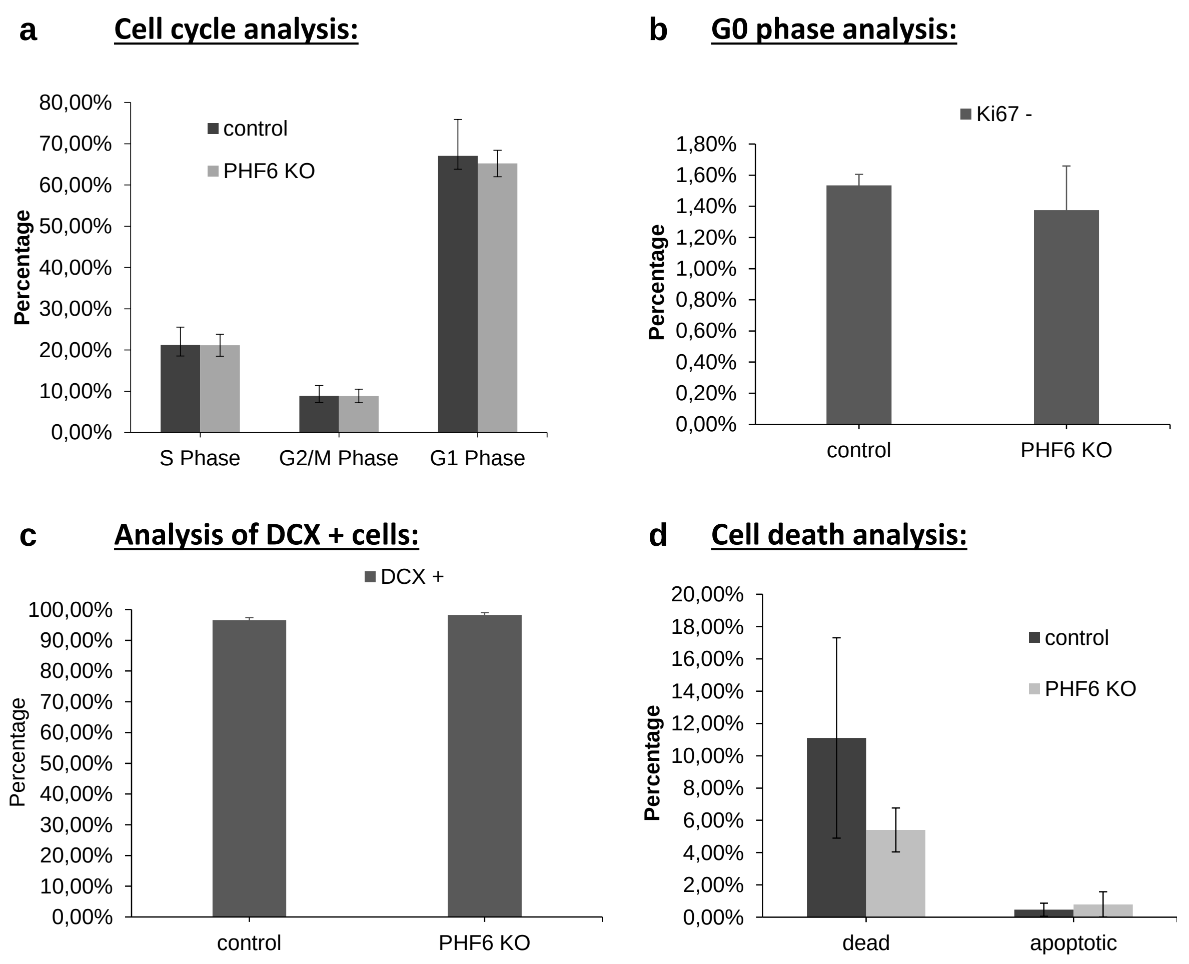

Supplementary Figure S8. Cell cycle and cell death analysis

(a) Day 10 differentiated control and *PHF6* KO SK-N-BE (2) cells were stained with EdU and FxCycle Violet Stain to separate cells based on their cell cycle phase. There was no difference detectable between the fraction of cells in the S phase, G2/M phase or G1 phase. (b) Day 10 differentiated control and *PHF6* KO SK-N-BE (2) cells were stained with Ki67 to determine the fraction of cells that are in G0 phase. Less than two percent of either control or KO cells were Ki67 negative, meaning they had entered G0 phase. (c) Day 10 differentiated control and *PHF6* KO SK-N-BE (2) cells were stained with DCX as a marker for differentiation. Nearly all control and KO cells were expressing DCX, no differences were detectable. (d) Day 10 differentiated control and *PHF6* KO SK-N-BE (2) cells were stained with SYTOX Green Nucleic Acid Stain and Annexin V to detect the percentage of dead and apoptotic cells. A low fraction was apoptotic or dead, and there was no difference between control and *PHF6* KO cells. Error Bars depict standard deviation.

Cell cycle analysis:

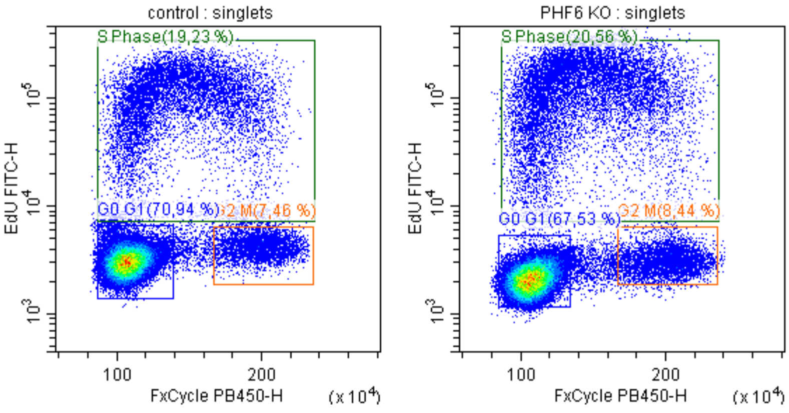

G0 phase analysis:

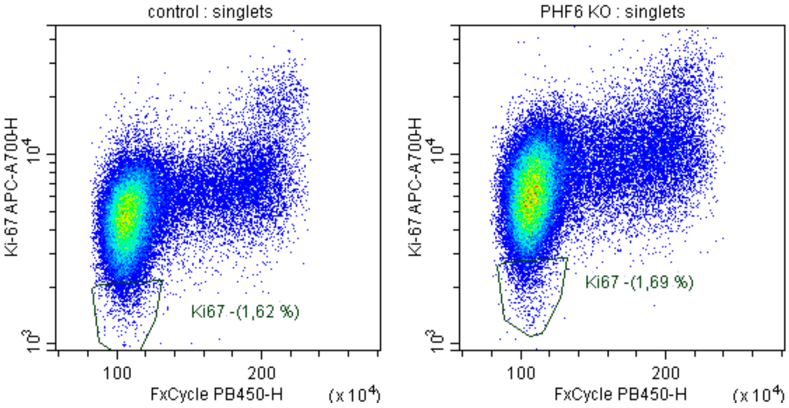

DCX +:

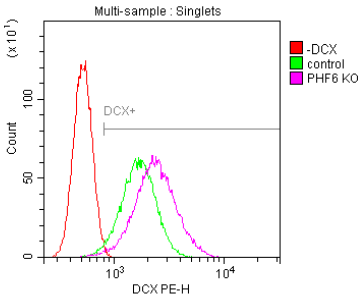

Cell death analysis:

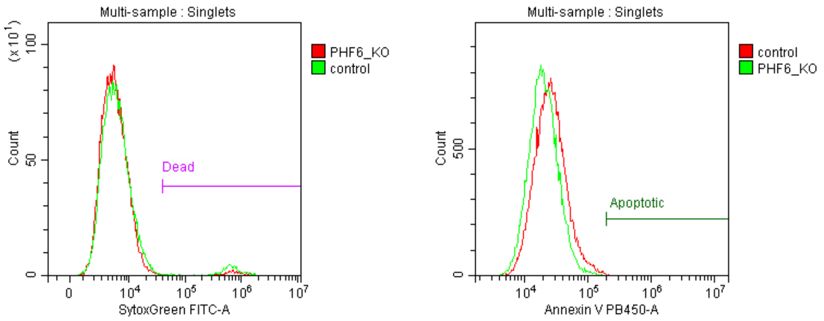

Supplementary Figure S9. FACS scans of cell cycle analysis and cell death analysis

Exemplary FACS scans of cell cycle analysis, G0 phase analysis, analysis of DCX positive (+) cells and cell death analysis. Analysis was performed using CytExpert (Beckman Coulter).

Supplementary Table S1. RNA sequencing (*PHF6* KO cells and *TCF4*-deficient patient blood), GO term analysis and CRISPR/Cas9 off targets (excel file)

Supplementary Table S2. crRNA sequences and potential off targets

crRNA_Exon2-fwd	CCTACAAGACAGCGCAAATGGTTTT
crRNA_Exon2-rev	CATTTGCGCTGTCTTGTAGGCGGTG
crRNA_Exon9-fwd	TACATTGAAAATATGTCACGGTTTT
crRNA_Exon9-rev	CGTGACATATTTTCAATGTACGGTG
Exon 2:	
MIT Guide Specifity Score	87
2 mismatches	1 off-target
3 mismatches	5 off-targets
4 mismatches	43 off-targets
Exon 9:	
MIT Guide Specifity Score	77
2 mismatches	1 off-target
3 mismatches	13 off-targets
4 mismatches	117 off-targets

Supplementary Material and Methods

Cell Cycle Analysis

Cell Cycle Assay was performed using the FxCycle Violet Stain Kit (invitrogen) and the Click-iT Plus EdU Flow Cytometry Assay Kit (invitrogen). These kits enable fluorescence-activated cell sorting (FACS)-based separation of cells depending on their cell cycle phase. As EdU incorporates into DNA during active DNA synthesis, cells can be separated into S-Phase (DNA synthesis phase) and not S-Phase. Additionally, as FxCycle Violet stain preferentially stains dsDNA, cells can be separated by their amount of DNA (G1-phase = 2C, G2/M-Phase = 4C). Taken together, cells can be separated in G1-, S- and G2/M-Phase. Assay was performed on day 10 differentiated SK-N-BE (2) cells following manufacturer's instructions. Additionally, cells

were stained with a fluorescent antibody against DCX (PE Mouse anti-Doublecortin, # 561505, BD Biosciences, 1:30) and an antibody against Ki-67 (Alexa Fluor 700 anti-human Ki-67 Antibody, #350530, BioLegend, 1:15) to determine the number of cells, which are in G0 phase, and evaluated using FACS sorting.

Cell Death and Apoptosis Analysis

Cell Death and Apoptosis Assay was performed using SYTOX Green Nucleic Acid Stain (Thermo Fisher Scientific) in combination with Annexin V. As Annexin V interacts with phosphatidylserine and is transported to the outside of the membrane only in apoptotic cells, the number of apoptotic cells can be determined. Additionally, as SYTOX Green Nucleic Acid Stain can only enter in cells with compromised membranes characteristic of dead cells, this can be used to differentiate between apoptotic and dead cells.

Assay was performed on day 10 differentiated SK-N-BE (2) cells. Briefly, cells were harvested, resuspended in Annexin V buffer containing Annexin V conjugate and SYTOX Green Nucleic Acid Stain, and incubated for 15 minutes. Annexin staining buffer was added and cells were analysed using FACS immediately.

Supplementary References

1. Liu, W., and Wang, X. (2019). Prediction of functional microRNA targets by integrative modeling of microRNA binding and target expression data. Genome Biology 20, 18.
2. Chen, Y., and Wang, X. (2020). miRDB: an online database for prediction of functional microRNA targets. Nucleic Acids Research 48, D127-D131.
3. Love, M.I., Huber, W., and Anders, S. (2014). Moderated estimation of fold change and dispersion for RNA-seq data with DESeq2. Genome Biology 15, 550.
